# Non-O1, Non-O139 *Vibrio cholerae* Bacteremia in an Autoimmune Pancreatitis Patient

**DOI:** 10.1155/2024/7219952

**Published:** 2024-05-03

**Authors:** Cheng-Jing Gu, Ming-Dong Ding, Da-Ming Zhou, Jie Li, Wen-Qing Yu, Yong-Lin Yang

**Affiliations:** ^1^Department of Pharmacy, Taizhou People's Hospital Affiliated to Nanjing Medical University, The Fifth Affiliated Hospital of Nantong University, Jiangsu 225300, China; ^2^Department of Infectious Diseases, Taizhou People's Hospital Affiliated to Nanjing Medical University, The Fifth Affiliated Hospital of Nantong University, Nantong, Jiangsu 225300, China; ^3^Department of Gastroenterology, Taizhou People's Hospital Affiliated to Nanjing Medical University, The Fifth Affiliated Hospital of Nantong University, Nantong, Jiangsu 225300, China

## Abstract

Over 200 different serogroups of *Vibrio cholerae* based on O-polysaccharide specificity have been described worldwide, including the two most important serogroups, O1 and O139. Non-O1/non-O139 *V. cholerae* serogroups generally do not produce the cholera-causing toxin but do sporadically cause gastroenteritis and extra-intestinal infections. Recently, however, bloodstream infections caused by non-O1/non-O139 *V. cholerae* are being increasingly reported, and these infections are associated with high mortality in immunocompromised hosts. We describe a case of non-O1/non-O139 *V. cholerae* bacteremia in a patient with autoimmune pancreatitis and stenosis of the intra- and extrahepatic bile ducts. The clinical manifestations of bacteremia were fever and mild digestive symptoms. The blood cultures showed *V. cholerae*, which was identified as a non-O1, non-O139 serogroup by slide agglutination tests and PCR. The bloodstream infection of the patient was likely caused by the consumption of contaminated seafood at a banquet. The patient recovered after the administration of a third-generation cephalosporin. Non-O1/non-O139 *V. cholerae* infection presents with or without gastrointestinal manifestations; close attention should be paid to the possibility of disseminated non-O1/non-O139 *V. cholerae* infection in high-risk patients.

## 1. Introduction

*Vibrio cholerae* is a species of Gram-negative bacteria with more than 200 serogroups [1]. *V. cholerae* strains belonging to serogroups O1 and O139 are responsible for cholera outbreaks [2]. In contrast, other *V. cholerae* strains, i.e., non-O1/non-O139 strains, generally do not produce the cholera toxin; these strains have occasionally been identified as invasive enteric pathogens in immunocompromised patients [2, 3]. The diverse putative virulence factors (such as hlyA, toxR, hap, rtxA, and T6SS) might be associated with the activities of non-O1/non-O139 *V. cholerae* strains to cause bacteremia [4]. However, those strains are highly heterogeneous and vary in genetics, antigenicity, and virulence properties. Autoimmune pancreatitis (AIP) is a relatively rare pancreatic disorder with an immune-mediated pathogenesis [4]. The cardinal features of AIP are abdominal pain, obstructive jaundice, and acute pancreatitis [5]. Steroid treatment is an effective choice for AIP patients, particularly for those with a high risk of relapse [6]. Here, we report a case of non-O1/non-O139 *V. cholerae* bacteremia in a man who was receiving maintenance therapy with oral corticosteroids after achieving remission from AIP.

## 2. Case Presentation

In June 2021, a 70-year-old man was admitted to Taizhou People's Hospital Affiliated with Nantong University, Jiangsu Province, China, due to a 1-day history of moderate fever with chills, appetite loss, nausea, and upper abdominal pain. Vomiting and diarrhea were not present. About three and a half years ago, the patient had developed AIP with obstructive jaundice, for which he underwent biliary stent implantation. In July 2019, after the jaundice was relieved, the stent was removed using endoscopic retrograde cholangiopancreatography. The patient was then administered oral prednisone 15 mg daily for maintenance treatment. One week prior to the current presentation, the patient attended a wedding banquet in a rural area, where he consumed seafood.

**Figure 1:**
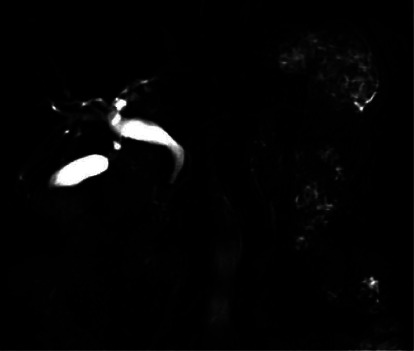
Magnetic resonance cholangiopancreatography shows diffuse intrahepatic and extrahepatic bile duct strictures with localized bead-like changes.

On admission, the patient was conscious and had stable vital signs. His physical examination was unremarkable, except for a slight epigastric tenderness in the right upper abdomen. The initial laboratory results are displayed in Table 1. His white blood cell count was 11.73 × 10^9^/L, with 81.1% neutrophils. The levels of C-reactive protein and procalcitonin were 54.30 mg/L and 0.42 pg/mL, respectively. Routine examinations of urine and stool samples, hepatic and renal functions, myocardial and pancreatic enzymes, tumor markers (including alpha-fetoprotein, carcinoembryonic antigen, and carbohydrate antigen (CA-19-9 and CA125)), humoral immunity (immunoglobulins A, G, M, and C4), and antibody profiles (including antibodies against mitochondrial M2, liver and kidney microsome type 1, hepatocyte cytoplasmic antigen type 1, and soluble liver antigen/pancreatic antigen) returned normal results, except for mild decreases in complement C3 and serum potassium, sodium, and chlorine levels. Serological tests for viral hepatitis (anti-hepatitis B virus and anti-hepatitis C virus) yielded negative results. No abnormalities in cardiac function or the heart valves were detected on echocardiography. Plain computed tomography scanning of the chest revealed slight chronic inflammation in both lungs, small nodules in the middle lobe of the right lung, and stenosis of intrahepatic bile ducts and common bile duct. Magnetic resonance cholangiopancreatography of the abdomen showed stenosis of the intra- and extrahepatic bile ducts, localized bead-like bile duct changes, uneven signals of the pancreatic head, and mild cholecystitis (Figure 1). Blood samples for culture examination were collected immediately after admission.

## 3. Diagnosis, Intervention, Follow-Up, and Outcome

The patient became afebrile after empirical treatment with ceftazidime (2 g, q8h) for 48 h. On day 3 of admission, routine blood cultures (two sets each in aerobic and anaerobic bottles) showed Gram-negative rods, which were identified as non-O1, non-O139 *V. cholerae* by slide agglutination tests (Ningbo Tianrun Biological Pharmaceutical Co. Ltd., Zhejiang Province, China). The identification result of bacterial species was confirmed via PCR by Sangon Biotech (Shanghai) Co., Ltd., China, and the Taizhou City Center for Disease Control and Prevention, Jiangsu Province, China. The antimicrobial susceptibility testing showed that the isolate was sensitive to most antibiotics, including piperacillin, ceftazidime, fluoroquinolones, aminoglycosides, and trimethoprim-sulfamethoxazole but was resistant to penicillin, cefradine, erythromycin, and clindamycin. After 10 days of antibiotic therapy, the patient's symptoms improved. Upon his insistence, the patient was discharged on day 10 and instructed to complete a 14-day course of oral levofloxacin 500 mg daily. Half a month later, the patient was called back and was found to have recovered.

## 4. Discussion

AIP is a rare, chronic fibro-inflammatory disorder of the pancreas, which responds to steroid treatment but requires long-term therapy for sustained remission [5, 7, 8]. To our knowledge, this is the first case report of an AIP patient who was diagnosed with bacteremia secondary to non-O1/non-O139 *V. cholerae*.

The non-O1/non-O139 strains of *V. cholerae* generally lack the two classical virulence factors (the cholera toxin and the toxin-coregulated pilus) of the O1 and O139 strains and sporadically cause gastroenteritis and extra-intestinal infections [9, 10]. However, serious life-threatening infection with those strains is being increasingly reported in immunocompromised patients [10, 11]. Non-O1/non-O139 *V. cholerae* infection is often associated with the exposure to contaminated seawater or seafood. As a resident of Taizhou City, which is located in the middle and lower reaches of the Yangtze River, our patient had easy access to marine products. The source of infection in our patient was very likely the seafood he consumed at the wedding banquet. His history of steroid treatment and consumption of possibly contaminated seafood accounted for this infection. Thus, the occurrence of non-O1/non-O139 *V. cholerae* infection in patients with chronic inflammatory pancreatitis highlights the importance of adequate attention in parenteral infections.

The common clinical manifestations of non-O1/non-O139 *V. cholerae* infection are fever, abdominal pain, nausea, vomiting, diarrhea, and other gastrointestinal manifestations; extra-intestinal presentation was also reported [2, 12]. A retrospective analysis of 32 cases from 2005 to 2019 in China showed that the clinical symptoms of non-O1/non-O139 *V. cholerae* bacteremia were fever, abdominal pain, and diarrhea [4]. In addition, most patients were male with common comorbidities, such as cirrhosis, malignant tumors, blood diseases, and diabetes. Importantly, the mortality rate was high, reaching 12.1% in the above review [4]. In addition, Maraki et al. reviewed 48 cases of non-O1/non-O139 *V. cholerae* bacteremia associated with skin and soft-tissue infections [13]. The authors found that most cases occurred in male patients with underlying illnesses, such as liver cirrhosis, chronic liver disease, and alcohol abuse [13]. Other studies have also described the following comorbidities as being risk factors for invasive infection with non-O1/non-O139 *V. cholerae*: liver cirrhosis, diabetes, pulmonary diseases, organ transplants, and hematological abnormalities [10, 14]. In our patient, the main clinical features were fever and mild digestive symptoms. It is possible that his illness was in its early stages, as it was the first day after onset. Although our patient was in remission and under oral corticosteroid therapy, the stenosis of the biliary tract secondary to AIP likely increased his susceptibility to non-O1/non-O139 *V. cholerae* infection.

Previous reports of study showed that the definite diagnosis is made by isolating *V. cholerae* via the culture and genotyping of clinical samples, including blood, stool, ascites fluid, the respiratory tract, bile, and others specimens [4]. Although the use of third-generation cephalosporins alone or in combination with tetracycline or fluoroquinolones is recommended, there is no clear evidence on which antimicrobial treatment is most suitable [14]. In our patient, there was no delay in diagnosis and treatment and no local transmission. He recovered after the prompt administration of a third-generation cephalosporin for 2 weeks. Thus, early detection and timely intervention can improve the prognosis of even high-risk individuals with non-O1/non-O139 *V. cholerae* infection. In the current study, a limitation was the lack of virulence gene detection. Further research on this case is needed.

## 5. Conclusion

Non-O1/non-O139 *V. cholerae* infection may vary widely, occasionally without gastrointestinal manifestations; close attention should be paid to the possibility of disseminated non-O1/non-O139 *V. cholerae* infection in high-risk patients.

## Figures and Tables

**Table 1:** Initial laboratory test results of an autoimmune pancreatitis patient with non-O1, non-O139 *Vibrio cholerae* septicemia.

Variable	Normal range	Value
Hematology		
WBC (×10^9^ cells/L)	4–10	11.73
NEU%	50–70	81.1
LYM%	20–40	9.0
MON%	3–10	9.4
PLT (×10^9^ cells/L)	100–300	206
Hemoglobin (g/L)	130–175	136
ESR (mm/h)	0–15	12
Serum biochemistry		
Total bilirubin (*μ*mol/L)	0–23	11.7
Direct bilirubin (*μ*mol/L)	0–6.8	2.9
ALT (U/L)	9–50	17
AST (U/L)	15–40	24
LDH (U/L)	120–250	201
CK (U/L)	0–164	44
Albumin (g/L)	40–55	32.0
CRE (*μ*mol/L)	57–111	45.0
Glucose (mmol/L)	3.9–6.1	4.01
Amylase (U/L)	35–135	48
Lipase (U/L)	5.6–51.3	24
Pancreatic amylase (U/L)	8–53	14
CRP (mg/L)	0–4	54.3
PCT (ng/mL)	0–0.1	0.402
Potassium (mmol/L)	3.5–5.3	3.13
Sodium (mmol/L)	137–147	128.1
Chlorine (mmol/L)	99–110	93.8
Immunoglobulin A (g/L)	0.7–4.0	1.64
Immunoglobulin G (g/L)	7.0–16.0	10.0
Immunoglobulin M (g/L)	0.4–2.3	2.00
Complement C3 (g/L)	0.9–1.8	0.73
Complement C4 (g/L)	0.1–0.4	0.36
Etiology		
Stool bacterial culture		Negative
Blood culture		Positive

WBC, white blood cell count; NEU%, percentage of neutrophils; LYM%, percentage of lymphocytes; MON%, percentage of monocytes; PLT, platelet count; ESR, erythrocyte sedimentation rate; ALT, alanine aminotransferase; AST, aspartate aminotransferase; LDH, lactate dehydrogenase; CK, creatine kinase; CRE, serum creatinine; CRP, C-reactive protein; PCT, serum procalcitonin.

## Data Availability

All data relating to this study are available upon request from the corresponding authors.

## References

[1] Unterweger D., Miyata S. T., Bachmann V. (2014). The *Vibrio cholerae* type VI secretion system employs diverse effector modules for intraspecific competition. *Nature Communications*.

[2] Ding Y., Hao J., Zeng Z., Liu J. (2022). Identification and genomic analysis of a *Vibrio cholerae* strain isolated from a patient with bloodstream infection. *Heliyon*.

[3] Arteaga M., Velasco J., Rodriguez S. (2020). Genomic characterization of the non-O1/non-O139 *Vibrio cholerae* strain that caused a gastroenteritis outbreak in Santiago, Chile, 2018. *Microbial Genomics*.

[4] Li X., Wu Y., Sun X. (2020). Non-O1/non-O139 *Vibrio cholerae* bacteraemia in mainland China from 2005 to 2019: clinical, epidemiological and genetic characteristics. *Epidemiology and Infection*.

[5] Okazaki K., Chari S. T., Frulloni L. (2017). International consensus for the treatment of autoimmune pancreatitis. *Pancreatology*.

[6] Khandelwal A., Inoue D., Takahashi N. (2020). Autoimmune pancreatitis: an update. *Abdom Radiol (NY)*.

[7] Matsubayashi H., Ishiwatari H., Imai K. (2019). Steroid therapy and steroid response in autoimmune pancreatitis. *International Journal of Molecular Sciences*.

[8] Culver E. L., Smit W. L., Evans C. (2017). No evidence to support a role for *Helicobacter pylori* infection and plasminogen binding protein in autoimmune pancreatitis and IgG4-related disease in a UK cohort. *Pancreatology*.

[9] Khandelwal A., Shanbhogue A. K., Takahashi N., Sandrasegaran K., Prasad S. R. (2014). Recent advances in the diagnosis and management of autoimmune pancreatitis. *American Journal of Roentgenology*.

[10] Laviad-Shitrit S., Sharaby Y., Izhaki I., Peretz A., Halpern M. (2018). Antimicrobial susceptibility of environmental non-O1/non-O139 *Vibrio cholerae* isolates. *Frontiers in Microbiology*.

[11] Zhang X., Lu Y., Qian H. (2020). <p>Non-O1, Non-O139 <em>*Vibrio cholerae*. *Infection and Drug Resistance*.

[12] Chen J., Huang J., Huang M. (2020). Two cases of septic shock with different outcomes caused by non-O1/non-O139 *Vibrio cholerae* isolates. *Journal of International Medical Research*.

[13] Maraki S., Christidou A., Anastasaki M., Scoulica E. (2016). Non-O1, non-O139 *Vibrio cholerae* bacteremic skin and soft tissue infections. *Infectious Diseases*.

[14] Lan N. P., Nga T. V., Yen N. T. (2014). Two cases of bacteriemia caused by nontoxigenic, non-O1, non-O139 *Vibrio cholerae* isolates in Ho Chi Minh City, Vietnam. *Journal of Clinical Microbiology*.

